# Neuroblastoma Cell Lines Contain Pluripotent Tumor Initiating Cells That Are Susceptible to a Targeted Oncolytic Virus

**DOI:** 10.1371/journal.pone.0004235

**Published:** 2009-01-21

**Authors:** Yonatan Y. Mahller, Jon P. Williams, William H. Baird, Bryan Mitton, Jonathan Grossheim, Yoshinaga Saeki, Jose A. Cancelas, Nancy Ratner, Timothy P. Cripe

**Affiliations:** 1 Division of Hematology and Oncology, Cincinnati Children's Hospital Medical Center, Cincinnati, Ohio, United States of America; 2 Division of Experimental Hematology, Cincinnati Children's Hospital Medical Center, Cincinnati, Ohio, United States of America; 3 Division of Pathology, Cincinnati Children's Hospital Medical Center, Cincinnati, Ohio, United States of America; 4 Physician Scientist Training Program, University of Cincinnati College of Medicine, Cincinnati, Ohio, United States of America; 5 Graduate Program of Molecular and Developmental Biology, University of Cincinnati College of Medicine, Cincinnati, Ohio, United States of America; 6 Dardinger Laboratory for Neuro-Oncology and Neurosciences, Department of Neurological Surgery and Comprehensive Cancer Center, The Ohio State University, Columbus, Ohio, United States of America; Karolinska Institutet, Sweden

## Abstract

**Background:**

Although disease remission can frequently be achieved for patients with neuroblastoma, relapse is common. The cancer stem cell theory suggests that rare tumorigenic cells, resistant to conventional therapy, are responsible for relapse. If true for neuroblastoma, improved cure rates may only be achieved via identification and therapeutic targeting of the neuroblastoma tumor initiating cell. Based on cues from normal stem cells, evidence for tumor populating progenitor cells has been found in a variety of cancers.

**Methodology/Principal Findings:**

Four of eight human neuroblastoma cell lines formed tumorspheres in neural stem cell media, and all contained some cells that expressed neurogenic stem cell markers including CD133, ABCG2, and nestin. Three lines tested could be induced into multi-lineage differentiation. LA-N-5 spheres were further studied and showed a verapamil-sensitive side population, relative resistance to doxorubicin, and CD133+ cells showed increased sphere formation and tumorigenicity. Oncolytic viruses, engineered to be clinically safe by genetic mutation, are emerging as next generation anticancer therapeutics. Because oncolytic viruses circumvent typical drug-resistance mechanisms, they may represent an effective therapy for chemotherapy-resistant tumor initiating cells. A Nestin-targeted oncolytic herpes simplex virus efficiently replicated within and killed neuroblastoma tumor initiating cells preventing their ability to form tumors in athymic nude mice.

**Conclusions/Significance:**

These results suggest that human neuroblastoma contains tumor initiating cells that may be effectively targeted by an oncolytic virus.

## Introduction

Neuroblastoma is the most common extracranial solid tumor found in children, accounting for 8–10% of childhood cancers. The median age of diagnosis is 17.3 months, with 40% of patients diagnosed as infants and 98% by 10 years of age [1], [2]. Most children with low or intermediate risk neuroblastoma achieve remission via a combination of surgery, radiation, and chemotherapy, and some children with high risk disease also benefit from megadose chemotherapy with autologous hematopoietic stem cell rescue [3]. Unfortunately, due to therapy-resistant relapse, long term survival in high risk cases is <50%. Risk factors indicative of poor prognosis include age >18 months, higher stage of the disease, *MYCN* amplification, and unfavorable histology [4].

It has been hypothesized for many cancers that tumor cells responsible for failures in long-term remission exhibit stem cell properties. Considerable evidence supports the hypothesis that leukemias arise from stem or progenitor cells, in an analogous fashion to hematopoiesis [5]. Although hematopoiesis is the most thoroughly studied stem cell system, many other organs are maintained by locally resident stem cells characterized by asymmetric cell division and multi-lineage differentiation [6]. Reports have shown that cancer cells expressing stem markers exhibit chemoresistance, thus supporting the notion that undifferentiated cells are responsible for clinical relapse [7].

There is now mounting data supporting the cancer stem cell theory for solid tumors [8]. Historically researchers have injected >10^6^ tumor cells to create xenograft models, but recent reports have identified sub-populations requiring as few as 10–200 cells to form tumors [9], [10], [11], [12], [13]. Tumor formation may be analogous to the development of normal tissues in that stem cells generate an organ with cellular diversity in marker expression, differentiation, therapeutic resistance and metastatic potential. Autonomous growth in serum-free media, as non-adherent neurospheres, is thought to be a surrogate marker of neural and malignant progenitors [9], [14], [15], [16], [17], [18]. Identification of normal and tumorigenic stem cells has also been studied by side population analysis in a technique that exploits the increased drug efflux capacity of stem cells [19], [20], [21], [22], [23], [24]. Highly tumorigenic cells often express CD133, a five transmembrane domain glycoprotein also expressed by untransformed hematopoietic and neural progenitors [11], [13], [23], [25]. CD133 expressing glioblastoma cells were significantly resistant to chemotherapeutic agents compared to cells not expressing this marker [26]. Other cancer stem cells have been described to express markers including CD20 and CD44^+^CD24^−^/lineage low for melanoma and breast cancer stem cells, respectively [10], [12]. Cancer cell subpopulations based only upon marker expression are likely to be inaccurate due to antigenic plasticity and therefore studies based upon functional assays are likely more reliable to identify tumor stem cells.

The prognostic significance of cellular heterogeneity of the neural crest lineage cells in neuroblastoma has begun to be described [27]. A sub-population of so-called intermediate (I-type) neuroblastoma cells showed increased tumorigenicity and patients whose tumors contained a higher percentage of these cells had increased relapse [28]. Interestingly, these cells expressed CD133 and showed asymmetric cell division [28], [29]. Other studies revealed that neuroblastoma cells express neural precursor markers including CD34, ABCG2, and nestin [7], [16], [18], [30]. Further evidence that neuroblastoma is a stem cell disease is the finding of side populations in 65% of primary neuroblastoma samples [7]. These data suggest that further characterization of neuroblastoma stem cells is warranted and such studies may be critical for improvement of anti-cancer therapeutics.

Tumor-targeted viral therapies have progressed rapidly to clinical trials in recent years [31], [32]. These biologic therapeutics demonstrate widespread tumor tropism [33]. We and others have shown neural tumors are sensitive to oncolytic Herpes simplex virus (oHSV) mutants and further hypothesized that these viruses may effectively destroy drug-resistant neuroblastoma tumor initiating cells [34], [35], [36], [37], [38], [39]. Herein we identified and isolated populations of human neuroblastoma cells that expressed CD133 and grew as clonal spheres. Tumorsphere-derived cells showed doxorubicin resistance and multi-lineage differentiation. CD133 expressing cells demonstrated increased tumorsphere forming ability and tumorigenicity in athymic nude mice. Finally, a nestin-targeted oHSV effectively replicated within and killed neuroblastoma tumor initiating cells as measured by prevention of tumorigenicity.

## Materials and Methods

### Cells and viruses

Vero and human neuroblastoma (LA-N-5, IMR-32, SKNBE(2), CHP-134, SHSY5Y, SKNSH, CHLA-20, CHLA-79) cells were gifts from Thomas Inge (Cincinnati Children's Hospital Medical Center, Cincinnati, OH) and Robert Seeger (Children's Hospital of Los Angeles, Los Angeles, CA); their origins and culture conditions have been described [40], [41]. MYCN status was confirmed in each cell line by fluorescence in situ hybridization (data not shown). rQLuc and rQNestin34.5 were created using a BAC based approach and have been described [42], [43]. Tumorsphere-media consisted of a 50∶50 mix of F12 and DMEM (Invitrogen, Carlsbad, CA), supplemented with 20 ng/ml EGF (R&D systems, Minneapolis, MN), 40 ng/ml bFGF (R&D systems), 1% B27 and N2 supplements (Invitrogen), 2 µg/ml heparin (Sigma, St. Louis, MO), 0.1 mM β-mercaptoethanol (Sigma) and 1× antibiotic/antimycotic (Mediatech, Herndon, VA). Tumorspheres were dissociated using non-enzymatic dissociation solution (Sigma) and strained over a 40 µM filter. For tumorsphere formation assays erlotinib at 10 µM or L685,458 (Sigma) at 1 mM were utilized in DMSO at a final concentration 0.1%. Cell viability and HSV replication assays were performed as previously described [39].

### Flow cytometry and side population (SP)

100, 10 or 1 cell(s) were plated in 150 µl of neurosphere media in 96-well dishes via FACS Vantage. Side population was performed by staining cells at 1×10^6^/ml with 5 µg/ml Hoechst 33342 (Sigma) +/−verapamil at 75 ng/ml (Sigma) for 45–60 min at 37°C. Cells were pelleted and stained with propidium iodide (1 µg/ml) prior to analysis. Cell analysis and purification was performed using a FACSVantage SE equipped with UV and 488 excitation lasers and DIVA software. Hoechst excitation and emission were performed as described [44]; the UV laser was set to 50 mW and pressure was adjusted to 13 psi. Quantification of cell markers were performed by FACSCaliber using anti-CD133 (Miltenyi Biotech, Gladbach, Germany), and ABCG2 (Stemcell Technologies, Vancouver, Canada), anti-CD334 (BD Biosciences, San Jose, CA), and anti-nestin (R&D Systems, Minneapolis, MN), according to the manufacturers' instructions, with cut-off gates based on isotype controls. For the nestin studies, cells were first permeabilized using the intracellular reagents protocol (R&D Systems).

### Electron microscopy

Uninfected and rQNestin34.5-infected tumorspheres were fixed in glutaraldehyde overnight. Scanning EM samples were sputter coated with a Gold-Palladium alloy, sectioned and viewed using a Hitachi Model S-3000N Scanning EM. Transmission EM samples were heavy metal stained with Uranyl Acetate and Lead Citrate, sectioned and viewed using a Hitachi Model H-7600 Transmission EM. Sputter coater was a Denton Vacuum Desk IV.

### Differentiation and staining

Clonal spheres were fixed (4% PFA overnight), cryoprotected with 20% sucrose for 12 h, embedded in OCT and sectioned at a thickness of 12 µM. Tumorsphere sections were incubated with rabbit anti-nestin (Chemicon International, Temecula, CA) at 1∶200 dilution followed by goat anti-rabbit FITC (Jackson Immunoresearch, West Grove, PA). For differentiation experiments, clonal spheres were dissociated and plated into chamber slides coated with poly-lysine and fibronectin (R&D Systems). Cells were grown in media as per Williams et al. [45] containing 10% serum +/−the following factors: neurogenic, βFGF 20 ng/ml for 5–7 days then 10 ng/ml BDNF, NGF, and NT3 for 7 days; gliogenic, forskolin 2 µM, β-heregulin 5 ng/ml, insulin 10 ng/ml; or fibroblastic, βFGF20 ng/ml and TGF-β 10 ng/ml. Cells were fixed and stained with rabbit anti-neurofilament-M (1∶200, Chemicon), mouse anti-GFAP (1∶500, Chemicon), rabbit anti-S100β (1∶2000, Chemicon) and mouse anti-SMA (1∶500, Sigma), followed by host-appropriate FITC or TRITC secondary (Jackson Immunoresearch) and co-stained with DAPI (Sigma). Images were taken using a Zeiss inverted fluorescence microscope and Openlab Software.

### *In vivo* models

Experiments were approved by the CCHMC Institutional Animal Care and Use Committee. 5,000 CD133^+^ or CD133^−^ neuroblastoma cells in 30% Matrigel (BD Biosciences) were injected subcutaneously in 5–6 week old female athymic mice (Harlan, Indianapolis, IN). To determine if oHSV infection could prevent tumor formation, cells were infected ex vivo at 5 plaque forming units per cell, incubated for 5 hrs, mixed with 30% Matrigel (BD Biosciences) and injected subcutaneously in 5–6 week old female athymic mice (Harlan, Indianapolis, IN). Tumor development was monitored 3 times per week and volume calculated by V = (L×W^2^)×π/6.

### Statistical Analysis

Comparison between two means was performed with an unpaired student's *t* test and more than two means by ANOVA. For small groups comparing tumor formation frequency, Fisher's Exact Test was used. All statistics were done using SPSS13.0.

## Results

### Neuroblastoma cell lines show evidence of a cancer stem cell

We surveyed a panel of neuroblastoma cell lines for subpopulations by assessment of stem cell marker (CD34, CD133, Nestin) expression, the presence of a verapamil sensitive side population and the ability to form clonal spheres. All cell lines expressed stem cell markers and side populations were detected in most (Table 1). Half of the lines readily formed tumorspheres when plated at clonal density in serum-free neural stem cell conditions. Interestingly, this result seemed to correlate with MYCN amplification (Table 1). Tumorsphere-formation, cell surface marker expression and side-population cells were further investigated using the LA-N-5 cell line as it showed MYCN amplification, contained CD34/CD133/nestin expressing cells and formed clonal tumorspheres.

**Table 1:** Stem cell-like characteristics of human neuroblastoma cell lines.

Cell Line	*MYCN* ^amp^	%CD34+	%CD133+	%DP	%Side Pop	%Nestin+	Spere-forming
LA-N-5	+	3.4	33.9	2.0	1.0	88.6	Yes
SK-N-BE(2)	+	40.6	2.6	2.0	5.4	80.8	Yes
IMR-32	+	80.1	21.3	21.0	n.d.	52.4	Yes
CHP-134	+	27.8	3.2	2.4	2.5	38.2	Yes
SHSY5Y	−	21.3	1.6	1.3	n.d.	59.7	No
SK-N-SH	−	40.5	3.9	3.6	16.9	68.7	No
CHLA-20	−	5.1	2.4	0.7	11.0	49.3	No
CHLA-79	−	30.3	47.2	20.3	40.5	54.9	No

*MYCN* ^amp^, amplification of the *MYCN* gene.

DP, double positive for CD34 and CD133.

Side pop, presence of a verapamil-sensitive side population on flow cytometry with Hoescht stain.

n.d., not done.

### Neuroblastoma cells form tumorspheres at clonal densities

**Figure 1:**
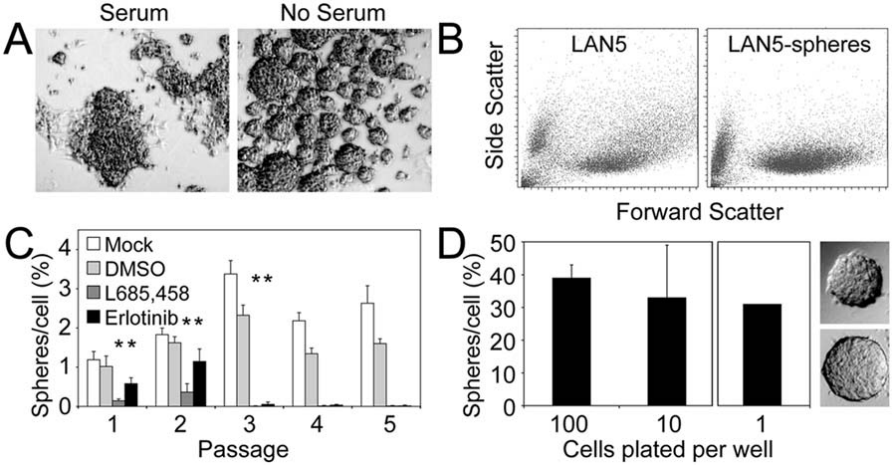
Neuroblastoma cells form tumorspheres in serum-free media. (A) LA-N-5 neuroblastoma cells plated in serum supplemented media show adherent growth while those plated in serum-free media (with EGF and bFGF) formed non-adherent tumorspheres. (B) Dissociated bulk and tumorsphere cells were subjected to FACS analysis. Tumorsphere cells showed increased uniformity in complexity (low side scatter) compared to bulk cultured cells. (C) Tumorsphere formation over serial passage (plating at supra-clonal density) in neurosphere media alone or supplemented with a γ-secretase inhibitor (L685,458, 1 mM) or an EGFR inhibitor (erlotinib, 10 µM). The inhibitors were dissolved in DMSO, which was used as a negative control. (D) Plating of dissociated LA-N-5 tumorsphere cells at 100 or 10 cells per well showed consistent sphere-forming efficiency regardless of plating density; 30% of wells plated with a single cell contained a single sphere. Pictures show examples of clonally derived spheres.

When plated above clonal density (10 cells/µl) many spheres formed by 1 week (Fig. 1A). Spheres ranging in size from 40 to 300 uM in diameter were observed and when dense often coalesced. Compared with bulk cultured cells, sphere-derived cells showed more cellular homogeneity in scatter patterns suggesting an element of cell selection (Fig. 1B). Serial passage showed enrichment for sphere-forming ability (p<0.0001 for passage 1 vs. 2 and 2 vs. 3), followed by a plateau, likely due to super-clonal plating density and sphere coalescence (confirmed by live video microscopy) (data not shown), a feature of normal neurospheres [46]. Also similar to *bona fide* untransformed neurospheres [47], [48], serial passage of LA-N-5 tumorspheres was dependent upon both gamma secretase activity and epidermal growth factor receptor (EGFR) signaling (*p<0.003) (Fig. 1C). To confirm clonality in tumorsphere formation, neuroblastoma cells (LA-N-5, IMR-32, CHP-134) were plated at 100, 10 and a single cell per well, by FACS Vantage cell sorter. All three cell lines showed clonal tumorsphere formation. LA-N-5 tumorsphere forming efficiency appeared steady regardless of plating density, with 30–40% of cells capable of forming spheres when plated at 100 or 10 cells per well, and 30% of wells receiving a single cell showing clonally derived spheres (Fig. 1D). Therefore human LA-N-5 neuroblastoma cells grown in serum-free media exhibited γ-secretase and EGFR-dependent clonal growth as tumorspheres and were enriched via serial passage. These studies highlight the heterogeneic nature of these tumorspheres as only ∼30% of cells reformed secondary spheres.

### Tumorsphere ultrastructure reveals dynamic three-dimensional arrangement

**Figure 2:**
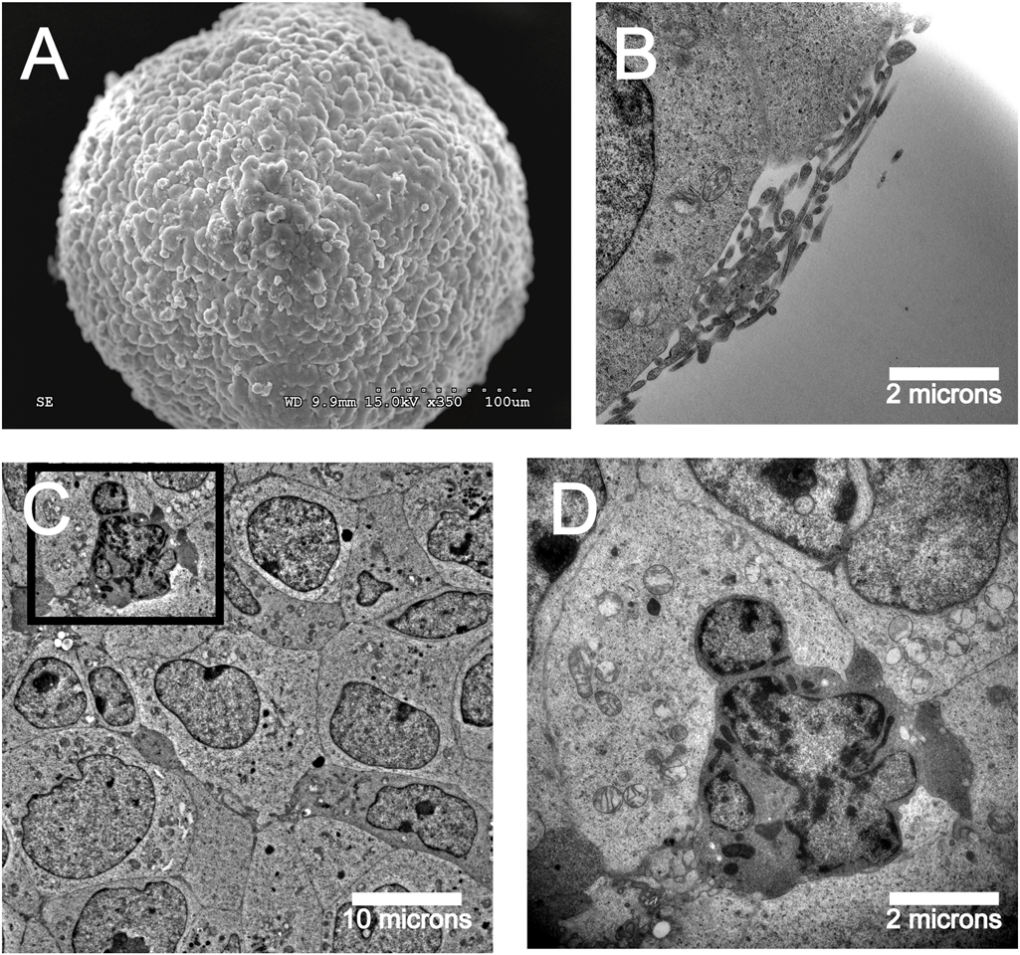
Ultrastructural characterization of neuroblastoma tumorspheres shows similarities to normal neurospheres. (A) Scanning EM of neuroblastoma tumorsphere shows a smooth and uniform surface. (B) Microvilli-like structures shown on the tumorsphere surface by transmission EM in cross-section. (C) Transmission EM shows tight packing of tumor cells in the tumorsphere core. (D) Apoptotic tumorsphere cell shows nuclear blebbing, chromatin fragmentation and mitochondrial swelling.

Electron microscopy (EM) on LA-N-5 neuroblastoma tumorspheres revealed similarities between tumorspheres and neurospheres. Scanning and transmission EM of the tumorsphere surface showed a smooth and uniform surface and revealed microvilli-like structures and apopotic cells (Fig. 2), which have also been reported in *bona fide* neurospheres [16], [46].

### Clonally-derived neuroblastoma tumorspheres show multi-lineage potential

**Figure 3:**
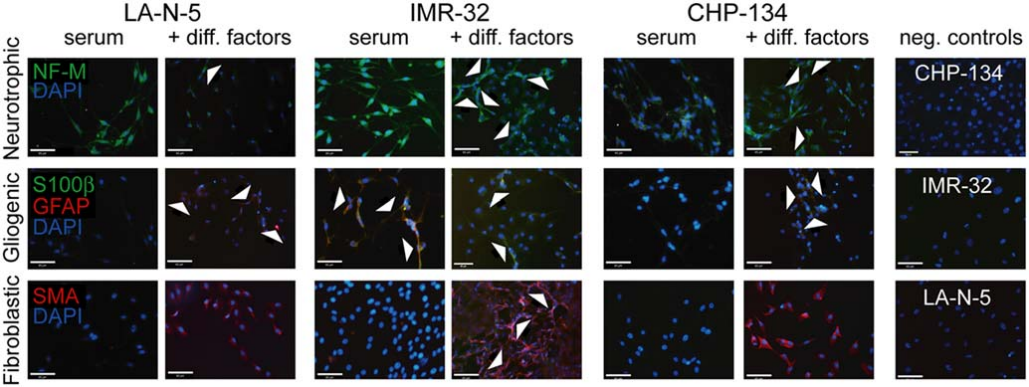
Clonally-derived neuroblastoma tumorsphere cells show multi-lineage differentiation. Clonally-derived tumorspheres from three different neuroblastoma cell lines were dissociated and cells were plated on poly-lysine and laminin coated chamber slides. Culture conditions were media containing serum alone or serum with neurotrophic factors (top row), gliogenic factors (middle row) or fibroblastic factors (bottom row) (except negative controls, which were serum alone without factors). Slides were stained with neurofilament-M (NF-M, green, top row), GFAP (red, middle row) S100β (green, middle row), or smooth muscle actin (red, bottom row); each were also co-stained with DAPI (blue). Negative control cultures were incubated without primary antibody and with secondary anti-mouse TRITC or anti-rabbit FITC. Arrows in the top and bottom rows indicate spindle-like cell extensions consistent with either neuronal or fibroblastic differentiation, while arrows in the middle row indicate positively stained cells. On close inspection of the GFAP/S100β stains, IMR-32 cells under serum conditions and CHP-134 cells supplemented with factors show co-staining with a mixture of green/red signals. Scale bars = 65 microns.

Clonally-derived tumorspheres (LA-N-5, IMR-32, CHP-134) were dissociated, plated on chamber-slides and grown in media containing serum alone or supplemented with neurogenic, gliogenic or smooth muscle fibroblastic growth factors. The cultures were stained with antibodies against various cytoskeletal and membrane proteins that have previously been validated as markers of neural lineage differentiation pathways, including neurofilament-M (NF-M) as a marker of neurogenic cell differentiation, S100β as a marker of Schwannian cell differentiation, glial fibulary acid protein (GFAP) as a marker of glial differentiation, and smooth muscle actin (SMA) as an indicator of fibroblastic differentiation [6], [45], [49], [50] (Fig. 3). All three cell lines grown in both serum and serum supplemented with neurotrophic factors expressed NF-M, though there was more evidence of neuronal-like morphologic changes in the supplemented cultures (Fig. 3, top row, best seen in IMR-32). There was a varied response of the different lines under gliogenic conditions: in LA-N-5 cells, GFAP but not S100β expression was clearly induced, in CHP-134 cells both appeared albeit only faintly, and in IMR-32 cells both were present at baseline but only S100β appeared after exposure to the factors (Fig. 3, middle row). Under fibroblastic conditions, all three cell lines showed marked induction of SMA (Fig. 3, bottom row). IMR-32 cells also showed a more elongated, spindly morphology under these conditions. These data suggest neuroblastoma cell lines harbor a subset of progenitor cells capable of multi-lineage differentiation.

### Tumorsphere cells are more resistant to doxorubicin and are enriched for CD133 expression

**Figure 4:**
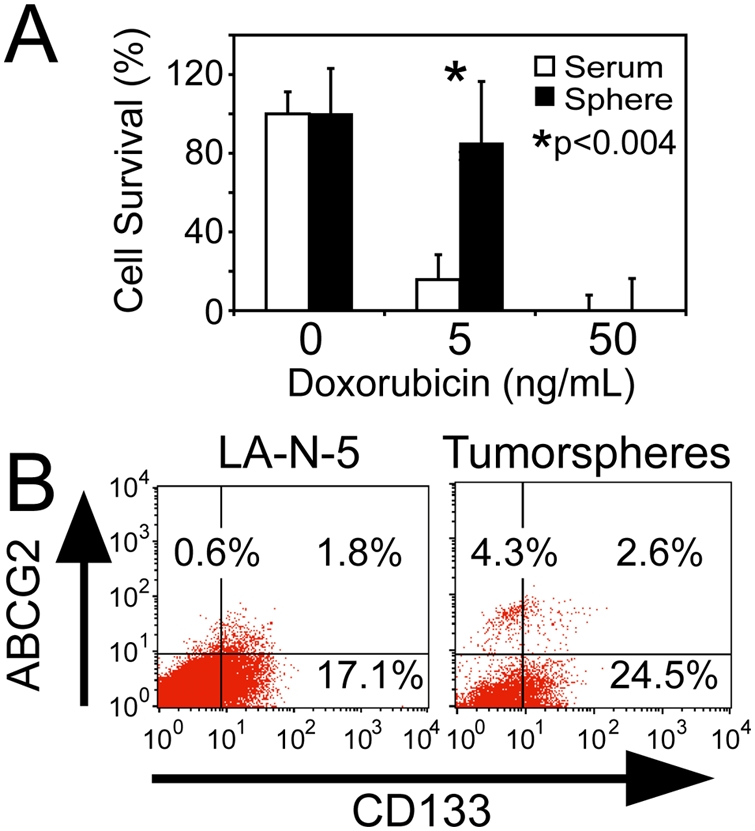
Neuroblastoma tumorsphere-derived cells are doxorubicin resistant and express ABCG2 and CD133. (A) LA-N-5 bulk and tumorsphere-derived cells were assessed for doxorubicin sensitivity by MTT assay at day 7. (B) Analysis for ABCG2 and CD133 expression in neuroblastoma cells grown as bulk culture or as tumorspheres.

We sought to determine if tumorsphere-derived cells were resistant to a cytotoxic chemotherapeutic, as might be expected for cancer stem cells [51]. Growth of LA-N-5 cells as bulk or tumorspheres in the presence of doxorubicin revealed that tumorsphere-derived cells exhibited relative resistance compared to bulk cells (Fig. 4A). Investigation of cell surface marker expression in bulk and tumorsphere-derived LA-N-5 cells revealed that tumorspheres are comprised of a more distinct subpopulation of cells expressing the drug efflux channel ABCG2, but the percentage of positive cells was only 2.9-fold increased, suggesting this is not likely to be a predominant mechanism responsible for the altered sensitivity. Cells exposed to doxorubicin also showed a small increase in cells expressing the CD133 (from 18.9% to 27.1%, Fig. 4B).

### Side population studies in neuroblastoma cells reveal asymmetric cell division

**Figure 5:**
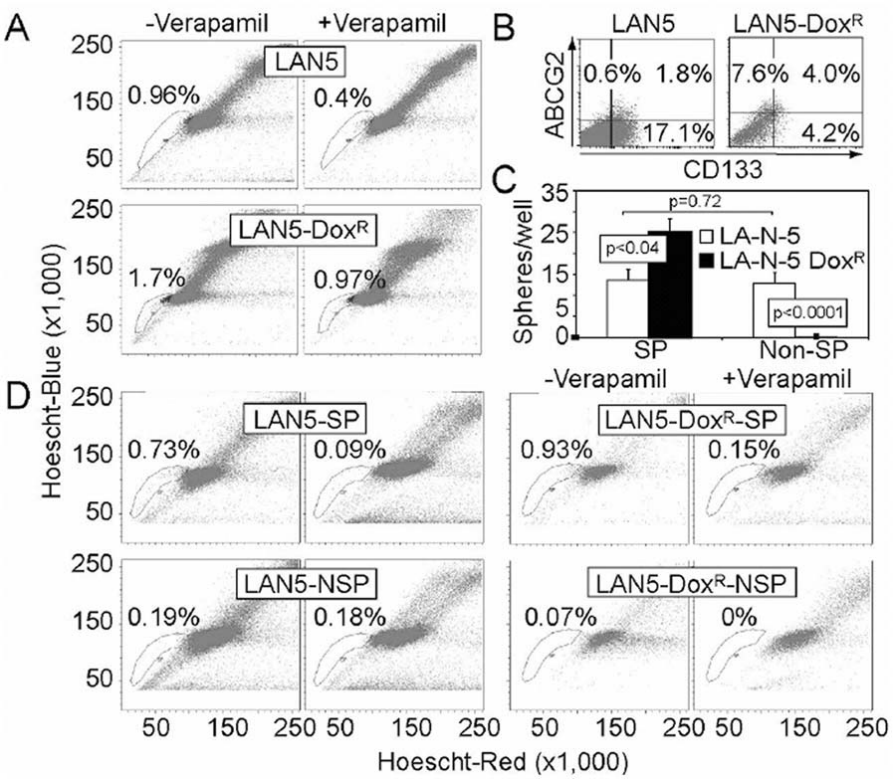
Side population analysis reveals asymmetric cell division of neuroblastoma cells. (A) Side population analysis using Hoechst 33342, +/−verapamil, of bulk LA-N-5 cells (LAN5) and LA-N-5 cells grown in doxorubicin (LAN5-dox^R^). (B) Analysis of ABCG2 and CD133 expression in LAN5 and LAN5-dox^R^ cells. (C) Sphere-forming efficiency of sorted SP and non-SP (NSP) cells from LAN5 and LAN5-dox^R^ cultures. (D) Sorted SP and NSP cells, from cultures of LAN5 and LAN5-dox^R^, were plated in serum containing media for 2 weeks and re-evaluated by side population, +/−verapamil. SP-derived cultures show regeneration of SP and NSP cells, while NSP-derived cultures showed only regeneration of NSP cells, not SP cells.

Expression of ATP-binding cassette transporters such as ABCG2 have been associated on flow cytometric analysis of Hoescht staining with the presence of a so-called “side population” (SP), thought to be enriched for stem cells in some tissues [19]. Thus, the presence of ABCG2 expression suggests that LA-N-5 cultures may contain an efflux channel-dependent SP. Indeed, approximately 1% of bulk LA-N-5 cells appeared in a verapamil-sensitive SP, which was nearly 2-fold enhanced following addition of doxorubicin to culture media (10 ng/ml for 7–12 days) (Fig. 5A). Most other tested neuroblastoma cell lines showed the presence of a verapamil-sensitive SP, ranging from 2.5–40.5% (Table 1). Characterization of cell surface expression of ABCG2 and CD133 in bulk and doxorubicin-treated LA-N-5 cultures revealed a 4.8-fold increase of cells expressing ABCG2 and a 2.2-fold increase in double-positive cells (Fig. 5B). Conversely, doxorubicin exposure significantly reduced the percentage of CD133 expressing cells, indicating that a large number of CD133 positive cells are indeed doxorubicin-sensitive. Further, these studies imply that CD133 expressing cells will reside in both the SP as well as the non-SP.

We next evaluated sphere-forming ability of SP and non-SP cells grown in neurosphere conditions for 2 weeks. Sorted SP and non-SP (NSP) cells from bulk cultured LA-N-5 showed no difference in sphere-forming ability, while SP and non-SP cells from LA-N-5 cells cultured with doxorubicin showed enrichment of sphere-forming ability for SP cells and total loss of sphere formation in the non-SP (Fig. 5C). Increased sphere-formation in the doxorubicin-cultured SP may reflect the doxorubicin-induced increase in cells expressing both CD133 and ABCG2, possibly cells with higher sphere-forming ability. Further, decreased sphere-formation in the doxorubicin-cultured non-SP cells may reflect the doxorubicin-mediated decrease in total cells expressing CD133.

As a test to determine asymmetric cell division, we cultured LA-N-5 SP and non-SP cells in serum containing media for 2 weeks and then re-analyzed them by SP analysis. Cultures initially derived from LA-N-5-SP cells regenerated both SP and NSP cells. In contrast, LA-N-5-NSP derived cultures only regenerated NSP cells (Fig. 5D). Similar findings of asymmetric cell division were observed in doxorubicin-treated LA-N-5 cultures. These data reveal that neuroblastoma cells are relatively resistant to doxorubicin, express CD133 and ABCG2 and are capable of asymmetric cell division.

### CD133 expressing cells show increased sphere and tumor formation

**Figure 6:**
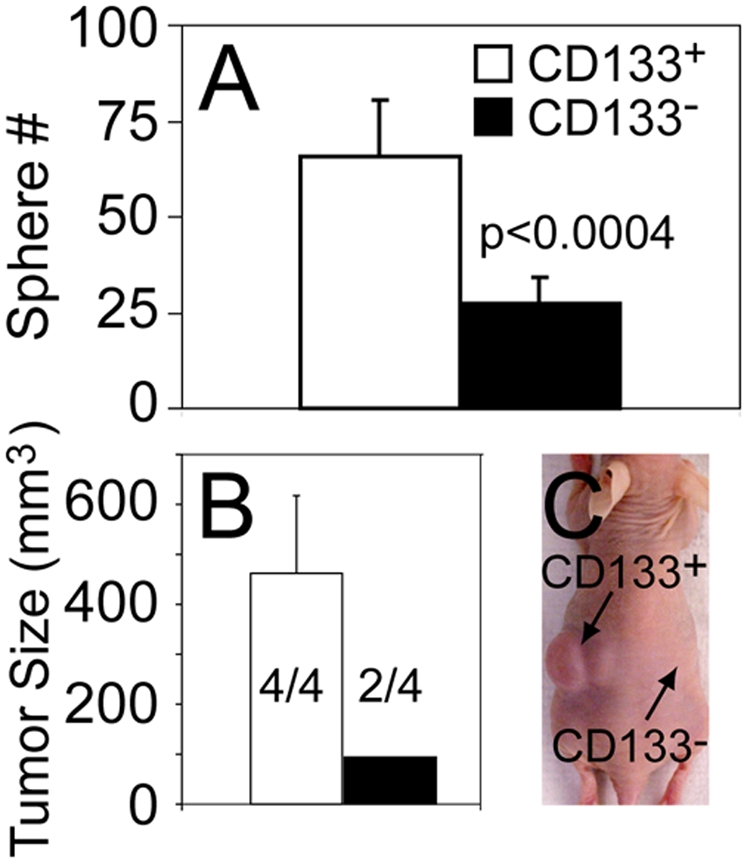
CD133 expressing neuroblastoma cells show increased sphere and tumor formation. Cells sorted for CD133 expression were assayed for (A) tumorsphere formation and (B) tumorigenicity following flank implantation of 5,000 cells in immunodeficient mice. Quantification of tumor volume and frequency were performed at 21 days post-inoculation. Numerators indicate number of flanks with tumors and denominators total number of flanks injected. (C) Image of mouse injected with CD133 expressing cells on the left flank and CD133 null cells on the right flank.

To determine if CD133 expressing cells showed functional differences from CD133 null cells, we sorted bulk LA-N-5 cells for CD133 and analyzed their ability to form tumorspheres and xenografts. At a plating density of 1.5 cells/µl, CD133 expressing cells exhibited increased sphere formation (Fig. 6A). Animals implanted with 5,000 CD133 expressing LA-N-5 cells demonstrated earlier tumor formation and larger size compared to CD133 null cells (p = 0.03), though the frequency of tumors did not reach statistical significance in this small experiment (p = 0.13 by Fisher's Exact test) (Fig. 6B, C).

### A transcriptionally-targeted oncolytic HSV kills neuroblastoma initiating cells

**Figure 7:**
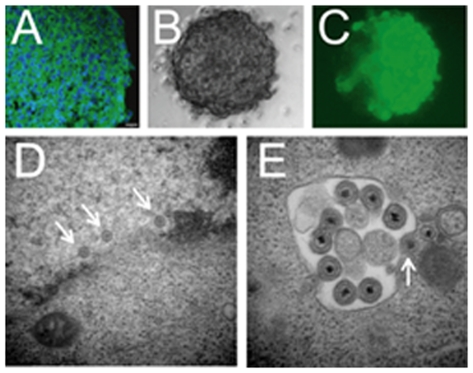
Neuroblastoma tumorspheres express nestin and are efficiently infected by oHSV. (A) Immunohistochemistry on tumorsphere cryosection for nestin (green) and DAPI co-stain (blue). Scale bar = 10 microns. (B, C) A neuroblastoma tumorsphere infected with rQNestin34.5 was imaged at 48 hours post-infection by (B) phase-contrast and (C) fluorescent microscopy for GFP. (D, E) rQNestin34.5-infected neuroblastoma tumorsphere at 48 hours post-infection evaluated by transmission electron microscopy showing viral nucleocapsids in the nucleus (arrows), and mature HSV particles in the cytoplasm in the process of acquiring their envelopes (arrows).

**Figure 8:**
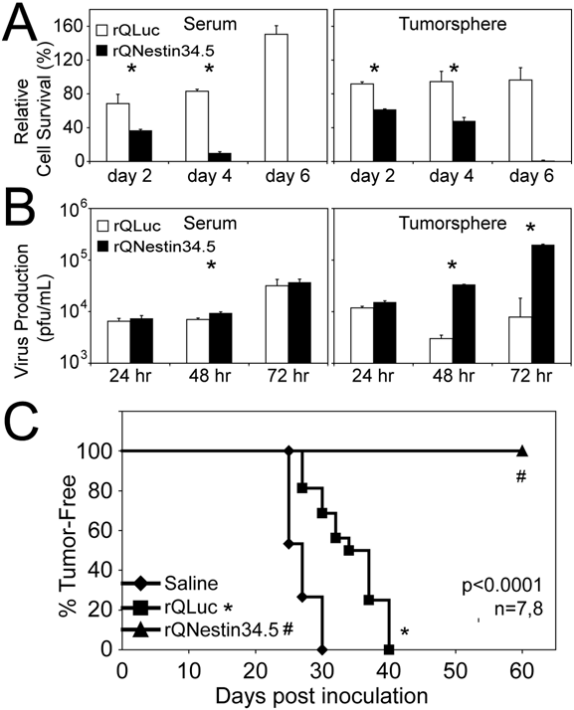
Neuroblastoma tumorsphere and tumor initiating cells are sensitive to a nestin-targeted oncolytic HSV. (A) Cytotoxicity assay on LA-N-5 bulk culture and tumorsphere-derived cells infected with rQNestin34.5 or the control virus, rQLuc. (B) Virus replication assay on LA-N-5 bulk culture and tumorsphere cells infected with rQNestin34.5 or rQLuc. (C) LA-N-5 cells were infected with oHSV *ex vivo*, followed by implantation into immunodeficient mice. Animals were followed over time for tumor development.

Based on nestin expression in primary neuroblastoma tumors and tumorspheres, we hypothesized that a nestin-targeted oHSV would be effective in killing both differentiated and tumor initiating neuroblastoma cells. rQNestin34.5 was initially created to target oHSV killing to nestin-positive brain tumor cells [43]. In this recombinant virus, expression of the HSV-1 neurovirulence gene (encoding γ_1_34.5) is driven by the nestin enhancer [43], and the virus remains attenuated in normal cells by a deletion in the virally-encoded large subunit of ribonucleotide reductase (ICP6). LA-N-5 neuroblastoma tumorspheres were positive for nestin by immunostain and all other tested neuroblastoma cell lines showed significant nestin expression (Fig. 7A and Table 1). Neuroblastoma tumorspheres were readily transduced by rQNestin34.5 (Fig. 7B, C). As predicted based on nestin expression, rQNestin34.5 caused significant cell death of both bulk and tumorsphere-derived LA-N-5 cells compared with the control virus, rQLuc (*p<0.05) (Fig. 8A). Virus replication was similar in bulk cultured LA-N-5 cells. In contrast, production of rQNestin34.5 was >10-fold increased compared with rQLuc in tumorsphere-derived cells (*p<0.05) (Fig. 8B). New virus production was confirmed to occur in tumorsphere cells by transmission EM of a virus-infected tumorsphere, demonstrating that virus isn't simply being produced exclusively in non-sphere cells present in the cultures. These cells showed viral nucleocapsids in the nucleus, particles in the cytoplasm acquiring their envelope and fully enveloped HSV particles (Fig. 7D, E).

Because the tumor initiating cells may represent only a small subpopulation of the cultures, we sought to determine if oHSV infection was truly affecting these cells. Cells were harvested, infected with oHSV *ex vivo* and injected subcutaneously into mice to detect tumorigenic cells. While rQLuc-treatment caused a significant delay in tumor formation compared with saline (from a mean of 25 to 35 days, p<0.001), treatment with rQNestin34.5 abolished tumor formation for >60 days (p<0.001, Fig. 8C), suggesting that the virus was capable of destroying the neuroblastoma tumor initiating cells present in the culture.

## Discussion

Human neuroblastoma is known for its striking tumor cell heterogeneity and ability to relapse. These features suggest that neuroblastoma may be a stem cell disease. Herein we describe studies using human neuroblastoma cell lines suggesting they contain subpopulations of cells with characteristics shared with normal neural stem cells. All cell lines tested contained cells that express markers previously described on neural stem cells such as CD133, ABCG2, and nestin. Half of the cell lines were capable of growing as “tumorspheres” in neural stem cell media, and three of three tested were capable of multi-lineage differentiation. One line tested further showed that the CD133 positive cells were enriched for tumorigenicity and that the spheres were enriched for a verapamil-sensitive side population, CD133 expression and doxorubicin resistance. Infection with a nestin promoter-directed oncolytc herpes virus prevented tumor formation, suggesting the tumor initiating cells were targetable by virotherapy.

We observed clonal growth in serum-free neurosphere media in four of eight neuroblastoma cells lines tested, which correlated with their MYCN amplification status. It is likely that MYCN expression plays a role in neuroblatoma stem cells given the recently described role of myc-regulated gene networks in generating induced pluripotent stem cells from somatic tissues [52] and the role of myc in the maintenance of both normal hematopoietic stem cells [53] and glioma cancer stem cells [54]. Growth of LA-N-5 neuroblastoma cells as non-adherent, nestin positive, multi-potent tumorspheres was dependent upon γ-secretase and EGFR signaling, similar to neural stem cells [17]. Tumorsphere-derived neuroblastoma cultures were enriched for stemness markers CD133 and ABCG2 in comparison to bulk-grown cells. Tumorsphere-derived cells showed relative resistance to doxorubicin.

As predicted, addition of doxorubicin to culture media of neuroblastoma cells increased the percentage of side population cells and correspondingly enriched cultures for ABCG2 expressing cells. Although CD133 positive and doxorubicin-sensitive cells were dramatically reduced in the presence of doxorubicin, those expressing ABCG2 and CD133 were enriched. These results demonstrate that the CD133 positive population is heterogeneous in expression of ABCG2 and that CD133 expression alone is not sufficient to identify neuroblastoma tumor initiating cells. CD133 as been identified as a marker of cancer stem cells in some models but not others, and its utility as a single marker of such cells is controversial [55]. Some studies have shown that CD133 expressing human cancer cells show chemoresistance [26], [56], [57]. In a tumorsphere formation assay, sorted SP and non-SP cells from bulk cultured LA-N-5 cells showed no difference, suggesting that sphere-forming cells exist in both. In contrast, SP cells from doxorubicin-treated LA-N-5 cultures showed enriched sphere forming ability compared with bulk culture SP cells, likely due to increased cells expressing ABCG2 and CD133. Further, non-SP cells from doxorubicin-treated LA-N-5 cultures lost the ability to form spheres, possibly due to doxorubicin-mediated destruction of ABCG2 null, CD133 expressing cells. These results demonstrate that doxorubicin treatment exerts multiple effects. First, it enriches the culture for cells with drug efflux ability and those able to form tumorspheres. Second, it diminishes the culture of cells lacking drug efflux ability but capable of forming spheres. Thus, side population or drug efflux status and sphere forming efficiency may be related at least partially via CD133 expression.

Asymmetric cell division is a key property of both normal and tumor stem cells. We observed that sorted SP-derived cultures could regenerate their non-SP counterparts, while non-SP-derived cultures could not. CD133 expressing cells showed increased ability to form tumorspheres and xenograft tumors in immunodeficient mice, suggesting increased tumorigenicity. Overall, these studies support the presence of cells with stem cell-like features in neuroblastoma and suggest that multiple criteria be utilized in parallel to further identify, isolate and characterize such cells.

Because cancer stem cells are believed responsible for tumor metastasis, escape from anticancer therapies and ultimately disease relapse, their therapeutic targeting is crucial [58], [59]. Small molecule inhibitors designed to target signaling pathways regulating stem cell renewal and maintenance may increase efficacy of traditional therapies. Hypothetical targets for future therapies include signaling via Notch, EGFR, Wnt, Hedgehog and bmi-1 [60]. Alternatively, it is conceivable that tumor initiating cells may be targeted via tumor cell-specific expression of stem cell surface markers using toxin-coupled antibodies. Combination of current chemotherapeutics with efflux blocking agents, such as calcium channel blockers, may enhance antitumor efficacy.

Another strategy for targeting cancer stem cells could be to utilize biologics. Oncolytic viruses are attractive anticancer therapeutics due to their ability to replicate *in vivo*, thereby amplifying the injected dose. These viruses have shown efficacy and safety in clinical trials [61], [62]. As drug-resistant tumor initiating cells have been reported to exist in a perivascular niche, intravenous administration of an oncolytic virus may be highly effective to reach this site [63]. In addition, because oncolytic viruses circumvent traditional chemotherapy resistance mechanisms, they have been thought to be ideal for targeting cancer stem cells [64]. As replication and toxicity of oncolytic viruses may be targeted via tumor specific promoters, we rationalized that such a virus could be targeted against neuroblastoma stem cells. In this study we demonstrated that a nestin-targeted oHSV efficiently infected, replicated and killed neuroblastoma tumorsphere cells. *Ex vivo* infection of neuroblastoma cells with a nestin-targeted oHSV resulted in death of tumor initiating cells as it prevented tumor development in animals. Our study used cell lines derived from neuroblastomas, so it will be important to verify our results using primary human samples. Spheres derived from bone marrow metastases of patients with neuroblastoma have been shown to be highly enriched for tumor initiating cells [9], suggesting these cultures may be useful for such validation studies.

Like several other cancers, it is becoming increasingly evident that neuroblastoma is a stem cell disease; thus, therapeutic targeting of cells that cause relapse is vital to improve patient outcome. Further study of neuroblastoma stem cells should reveal their roles in tumor initiation, progression and metastasis. As anticancer therapies incorporate anti-stem cell approaches, it will be important to ensure that these treatments do not adversely affect the function of normal tissue stem cells. Development of such innovative strategies to target cancer stem cell populations in human malignancies is likely to increase treatment success.
